# Lack of direct effect of adiponectin on vascular smooth muscle cell BK_Ca_ channels or Ca^2+^ signaling in the regulation of small artery pressure‐induced constriction

**DOI:** 10.14814/phy2.13337

**Published:** 2017-08-22

**Authors:** Rachael Baylie, Majid Ahmed, Adrian D. Bonev, David C. Hill‐Eubanks, Thomas J. Heppner, Mark T. Nelson, Adam S. Greenstein

**Affiliations:** ^1^ Division of Cardiovascular Sciences Faculty of Biology, Medicine and Health University of Manchester Manchester UK; ^2^ Department of Pharmacology University of Vermont Burlington Vermont

**Keywords:** Adiponectin, BK channel, calcium, perivascular adipose tissue

## Abstract

The aim of this study was to investigate mechanisms by which adiponectin influences vascular Ca^2+^ signaling, K^+^ channel activity and thus contractile tone of small arteries. Vasodilation to adiponectin was studied in mesenteric resistance arteries constricted with intraluminal pressure. Ca^2+^ signals were characterized using high speed confocal microscopy of intact arteries. Patch clamp investigated the effect of adiponectin on individual VSMC potassium (K^+^) channel currents. Adiponectin dilated arteries constricted with pressure‐induced tone by approximately 5% and the induced vasodilation was only transient. The dilation to adiponectin was reduced by pharmacological interruption of the Ca^2+^ spark/large conductance activated K^+^ (BK) channel pathway but from a physiological perspective, interpretation of the data was limited by the small effect. Neither Adiponectin nor the presence of intact perivascular adipose tissue (PVAT) influenced Ca^2+^ spark or Ca^2+^ wave frequency or characteristics. Studied using a perforated patch approach, Adiponectin marginally increased current through the VSMC BK channel but this effect was lost using the whole cell technique with dialysis of the cytoplasm. Adiponectin did not change the frequency or amplitude of Ca^2+^ spark‐induced transient outward currents (STOC). Overall, our study shows that Adiponectin induces only a small and transient dilation of pressure constricted mesenteric arteries. This vasodilatory effect is likely to be independent of Ca^2+^ sparks or direct BK channel activation.

## Introduction

Over the last two decades there has been a devastating surge in the cardiovascular complications of obesity. Many of these complications are attributable to obesity‐related hypertension, now acknowledged to be both a specific disease entity and also one of the leading worldwide causes of hypertension (Narkiewicz 2006). However, despite international consensus on the emergence and cardiovascular ramifications of obesity‐related hypertension, there is very little understanding of the cellular mechanisms by which weight gain leads to a rise in blood pressure. In this regard, one particular area increasingly of interest is the local vasodilatory effect of adipose tissue which surrounds small arteries, known as Perivascular Adipose Tissue (PVAT) (Yudkin et al. 2005). Studies of small arteries using wire myography have shown that the presence of intact PVAT attached to the vessel reduces agonist‐induced vasoconstriction (Dashwood et al. 2007; Gao 2007; Greenstein et al. 2009; Gollasch and Dubrovska 2004; Lohn et al. 2002; Dubrovska et al. 2004). The vasodilatory effect of the attached PVAT is not due to reduction in access of the agonist through the fat because the functional properties of PVAT are completely lost in both human and animal obesity (Greenstein et al. 2009; Marchesi et al. 2009). Given that up to 60% of all small arteries (mesenteric, subcutaneous, and skeletal) are surrounded by PVAT, the resultant global increase in resistance artery contractile tone caused by damage to PVAT vasodilatory function in obesity is likely to contribute to the development of obesity‐related hypertension (Gao 2007).

We have previously demonstrated that in human subcutaneous resistance arteries (internal diameter 250–300 *μ*m in diameter), a substantial component of the anticontractile effect of PVAT is due to release of adiponectin (Greenstein et al. 2009). Furthermore, a follow‐up study suggested that functional recovery of PVAT following bariatric surgery may be in part due to restoration of adiponectin in the PVAT itself (Aghamohammadzadeh et al. 2013). Adiponectin is a 30kD protein which is released exclusively from adipocytes, and traditionally has been studied in relation to its beneficial anti‐inflammatory and metabolic effects, thought to be mediated through activation of AMP Kinase (Iwabu et al. 2010; Yamauchi et al. 2007, 2002). Fesus and colleagues were the first group to show a vasodilation to adiponectin, demonstrating a robust effect of adiponectin at healthy human serum concentrations (3 *μ*g/mL) on wire mounted rat or mouse aorta or mouse mesenteric arteries preconstricted with serotonin (Fesus et al. 2007). The vasodilatory effect was independent of the endothelium and was blunted by 4‐aminopyridine, suggesting involvement of the vascular smooth muscle cell (VSMC) voltage gated potassium (*K*_v_) channel. Subsequently, two studies also using adiponectin at a dose of 3–5 *μ*g/mL suggested an alternative biophysical mechanism whereby adiponectin directly activates the large conductance calcium activated potassium (BK) channel. In these studies, blockade of the BK channel prevented both vasodilation to adiponectin of preconstricted mouse mesenteric arteries (Lynch et al. 2013) and the adiponectin‐induced hyperpolarization of mesenteric arteries (Lynch et al. 2013; Weston et al. 2013). Notably, however, the vasodilation to adiponectin has been very variable between the different studies, ranging from 6% (Lynch et al. 2013) to 67% (Fesus et al. 2007) even when comparing similar vascular beds. Furthermore, and in contrast to the earlier reports, a recent study has been unable to reproduce the vasodilation of preconstricted mouse aorta to adiponectin, even at higher doses (up to 15 *μ*g/mL) (Du et al. 2016), although in this study adiponectin did augment the vasodilation to acetylcholine. Perhaps consistent with this, Lynch et al. also observed that the vasodilation to adiponectin was enhanced when PVAT was left intact or when the endothelium was removed (Lynch et al. 2013), suggesting additional mechanisms.

Nevertheless were adiponectin able to influence BK channel function as previously suggested (Fesus et al. 2007; Lynch et al. 2013; Weston et al. 2013), elucidation of the underlying vasodilatory mechanism would have significant translational potential as a future therapeutic avenue in the treatment of obesity‐related hypertension. The vasodilatory capacity of VSMC BK channels is blunted in obesity and this phenomenon has been posited as a potential cause for the increased contractility of small arteries underlying obesity‐related hypertension (Nystoriak et al. 2014; Rusch Nov 2009). Furthermore, it is also established that obesity results in lower serum adiponectin levels (Merl et al. 2005) (Healthy human males: ~6–7 *μ*g/mL vs. Obese males: ~3–4 *μ*g/mL (Merl et al. 2005)). Therefore, given the previous work suggesting that adiponectin vasodilates small arteries via activation of the VSMC BK channel, we attempted to further clarify this adiponectin‐to‐BK channel hypothesis.

From a physiological perspective, BK channel function plays a key role in the regulation of myogenic tone, which is defined as the spontaneous constriction of resistance arteries in response to intraluminal pressure independently of contractile agonists. During pressure‐induced constriction, BK channels are activated by calcium (Ca^2+^) sparks; brief localized Ca^2+^ events from the VSMC sarcoplasmic reticulum (Perez et al. 2001). Ca^2+^ spark‐induced activation of the BK channel is manifested as spontaneous transient outward currents (‘STOC’) (Nelson et al. 1995). STOCs cause membrane hyperpolarization of the VSMC that subsequently initiates vasodilation and opposes the tonic contraction of myogenic autoregulation (Nelson et al. 1995; Wellman et al. 2002). Studies to date involving the vascular effects of PVAT and adiponectin on the BK channel are based on agonist‐induced constriction – interventions which significantly increase VSMC cytoplasm with Ca^2+^ through summation of waves (Wier and Morgan 2003). In this context, the effects of Ca^2+^ sparks on the BK channel and therefore STOCS are difficult to interpret, although the channel still has an important role (Hill et al. 1997). We therefore examined the vasodilation to adiponectin on arteries constricted with pressure‐induced constriction, examined the effect of adiponectin and PVAT on Ca^2+^ sparks in intact arteries using high speed confocal microscopy and corroborated our results with BK channel function measured using both the whole cell and perforated configurations of the patch clamp technique.

## Methods

### Materials

Ryanodine and globular adiponectin were purchased from Enzo Life Sciences (Farmingdale, NY). All other chemicals were obtained from Sigma‐Aldrich (St. Louis, MO). Adiponectin was used at a physiological concentration (5 *μ*g/mL), as described above.

### Tissue preparation and animal models

All animal procedures in this study were performed in accordance with institutional guidelines following approval by the University of Vermont Institute of Animal Care and Use Committee or in the UK in accordance with the UK Home Office Guidance on the Operation of the Animals (Scientific Procedures) Act 1986 and were approved by an institutional review committee. For the purposes of this study, we used wild‐type C57BL6 mice (Charles River). The mice used in the study were euthanized at the age of 3–4 months by intraperitoneal injection of sodium pentobarbital (150 mg/kg). The mesenteric bed was removed and kept in ice‐cold Hepes‐buffered physiological saline solution (HEPES‐PSS) with composition: 134 mmol/L NaCl, 6 mmol/L KCl, 1 mmol/L MgCl2, 2 mmol/L CaCl2, 7 mmol/L glucose, and 10 mmol/L HEPES with pH adjusted to 7.4 with 1 mol/L NaOH.

### Pressure myography

Third‐order mesenteric arteries (~130 *μ*m internal diameter) were dissected free of surrounding tissue and mounted on Borosilicate glass pipettes in an arteriograph chamber (Living Systems Instrumentation, St. Albans, VT). The proximal glass pipette was attached to a servo‐controlled pressure‐regulating device (Living Systems Instrumentation) while the distal pipette was blocked. After checking for leaks, arteries were pressurized to 80 mmHg for approximately 45 min in physiological saline solution (PSS) with composition: 119 mmol/L NaCl, 4.7 mmol/L KCl, 1.2 mmol/L KH_2_PO_4_, 1.2 mmol/L MgCl_2_, 2 mmol/L CaCl_2_, 7 mmol/L Glucose, 24 mmol/L NaHCO_3_, 2.3 mmol/L EDTA. PSS was warmed to 37°C and continuously gassed with Biological Gas (95% air and 5% CO_2_). Any artery exhibiting a leak was discarded and pharmacological protocols were only performed on arteries which exhibited spontaneous myogenic tone. When required, endothelium was removed using injection through the artery of an air bubble. Confirmation of endothelium denudation was performed by observing a lack of vasodilator response to acetylcholine (10^−5^ mol/L). Internal diameter was detected using a CCD camera and IonOptix edge‐detection software (Milton, MA). Arteries were allowed to develop spontaneous myogenic tone in circulating PSS (usually between 500 mL and 1 L). Once stable tone had developed we initially used a much reduced volume (25 mL) of circulating PSS to assess the vasodilatory properties of Adiponectin, the prohibitive cost of which prevented larger volumes from being used. However, using this protocol, we observed that the vasodilation to adiponectin was transient, although reproducible. We suspected that this was due to adherence of adiponectin to the plastic tubing, and were unable to technically modify the apparatus in order to reduce this effect. For the majority of the experiments therefore (and the data presented in the manuscript), the artery was mounted in a Living Systems self‐heating chamber and once myogenic tone had developed, the circulating PSS was stopped and the vessel allowed to acclimatize. Biological gas was applied to the chamber throughout the protocol (both with circulating and static PSS). The mounted arteries, typically exhibited a small vasodilation following the cessation of circulation, although this was not universal. Following stabilization of diameter, adiponectin was applied directly to the chamber. For the protocols with paxilline or ryanodine, the inhibitors were administered into circulating PSS prior to cessation of circulation. Endothelium denudation was achieved using the air‐bubble technique and confirmed by lack of response to acetylcholine. Ca^2+^‐free PSS was applied at the end of each experiment to determine passive diameter (by resumption of circulation). Myogenic tone is expressed as the difference between the vessel diameter in Ca^2+^‐containing and Ca^2+^‐free solutions expressed as a percentage of the diameter in Ca^2+^‐free conditions (i.e., change in diameter to adiponectin/diameter of artery in Ca^2+^‐free solution). Dilation to agonists was expressed as a change in diameter as a percentage of passive diameter in Ca‐free solution, in order to account for variation in arterial diameter and also to prevent artificial interpretation of diameter changes following the arterial constriction to paxilline or ryanodine.

### VSMC Ca^2+^ imaging

Third‐order mesenteric arteries were placed in HEPES‐PSS containing 10 *μ*mol/L fluo‐4 AM and 0.05% Pluronic acid for 1 h at room temperature. This was followed by a 30‐min wash in HEPES‐PSS. Arteries were subsequently mounted on glass pipettes in an arteriography chamber (as described above), pressurized to 80 mmHg in PSS (composition as above) and gassed with 95% O_2_ and 5% CO_2_. Following 30 min of equilibration in the PSS, fluorescence images were acquired using a Noran laser‐scanning inverted confocal microscope (Noran Instruments, Middleton, WI) and a 60× water immersion objective (final magnification, 600×; NA1.2) which was attached to a Nikon Eclipse TE‐2000U microscope. The fluo‐4–loaded arteries were excited by illuminating at 488 nm using a solid‐state laser, and fluorescence emission was collected above 510 nm. Images (256 × 256 pixels or 131 × 131 *μ*m) were recorded every 18.9 msec (53 images per second). Ca^2+^ sparks were identified using custom‐written software (Sparkan; Dr A. Bonev) which detects temporally delineated increases in fractional fluorescence (*F*) greater than 1.3 above baseline fluorescence levels (*F*_o_) in a defined area of interest 1.1 × 1.1 *μ*m (5 × 5 pixels); Ca^2+^ increases are expressed as *F*/*F*_o_. Arterial Ca^2+^ waves were identified manually as areas of increased Ca^2+^ which spread throughout the cell. When studying Ca^2+^ events in arteries with intact PVAT, adipose tissue was trimmed from one side of the artery in order to advance the objective as closely as possible to the vascular smooth muscle cell. The area studied was therefore immediately adjacent to the residual PVAT.

### Patch clamp electrophysiology

Mesenteric arteries were digested in dissociation solution (80 mmol/L Na glutamate, 55 mmol/L NaCl, 10 mmol/L HEPES pH 7.3, 6 mmol/L KCl, 2 mmol/L MgCl_2_, 0.5 mg/mL human albumin, 50 *μ*mol/L CaCl_2_ and 10 mmol/L glucose) containing Papain (Worthington, MA) 1 mg/mL and Dihdroerythritol 0.5 mg/mL for 25 min at 37°C. Following this, 0.5 mg/mL elastase, 2 mg/mL collagenase (Type 4) and 1 mg/mL trypsin inhibitor were added to the dissociation solution and the digestion was continued for a further 10 min. The artery was then dissected into segments and gently triturated in Ca^2+^‐free dissociation solution. The isolated smooth muscle cells, still in Ca^2+^‐free dissociation solution, were then transferred to the electrophysiology chamber and left for 30 min to adhere to the bottom (a glass coverslip). Outward K^+^ currents were measured using the whole‐cell, perforated‐patch configuration of the patch‐clamp technique using an Axopatch 200A amplifier (Axon Instruments, Union City, CA). Amphotericin (200 *μ*g/mL) was used to enable perforation. Composition of the extracellular solution: 134 mmol/L NaCl, 6 mmol/L KCl, 1 mmol/L MgCl_2_, 2 mmol/L CaCl_2_, 7 mmol/L glucose, and 10 mmol/L HEPES. (pH 7.4). Composition of the pipette solution: 110 mmol/L potassium aspartate, 30 mmol/L KCl, 10 mmol/L NaCl, 1 mmol/L MgCl_2_ and 10 mmol/L HEPES (pH 7.2). Patch clamp protocols were also performed using the traditional whole cell configuration, in order to delineate whether adiponectin could mediate effects once there had been dialysis of the cytoplasm through the pipette. For these protocols, the pipette solution contained 110 mmol/L K Aspartate, 30 mmol/L KCl, 10 mmol/L NaCl, 1 mmol/L MgCl2, 10 mmol/L HEPES, 5 mmol/L EGTA, 2.5418 mmol/L CaCl2 (200 nmol/L free Ca^2+^ at pH 7.2 at 25°C). For all experiments, currents were filtered at 1KHz and digitized at 4 kHz. Protocols to measure Kv and BK channel currents were performed in the presence of ryanodine (20 mmol/L). The BK channel current was estimated as the decrease in current following application of paxilline (1 *μ*mol/L). Voltage step protocols started from a holding potential of −50 mV prior to 250 msec steps from −40 mV to +50 mV with 10 mV increments (10 steps, for protocol see Figure 3D). Voltage ramps measured current secondary to a linear increase in voltage from −100 mV to +100 mV over 250 msec with a holding potential of −50 mV (Figure 3E). Spontaneous transient outward currents (STOCs) were measured in extracellular solution (composition above) without ryanodine. Currents were analyzed using Clampfit.

### Statistical analysis

All values are presented as mean and standard error of the mean. Students *t*‐test (unpaired, two tailed), nonparametric (Wilcoxon signed rank test) analysis and multiple points ANOVA were used as appropriate. *P* < 0.05 was considered to be significant.

## Results

### Adiponectin‐induced dilation of pressure constricted mesenteric arteries is small and nonsustained

**Figure 1:**
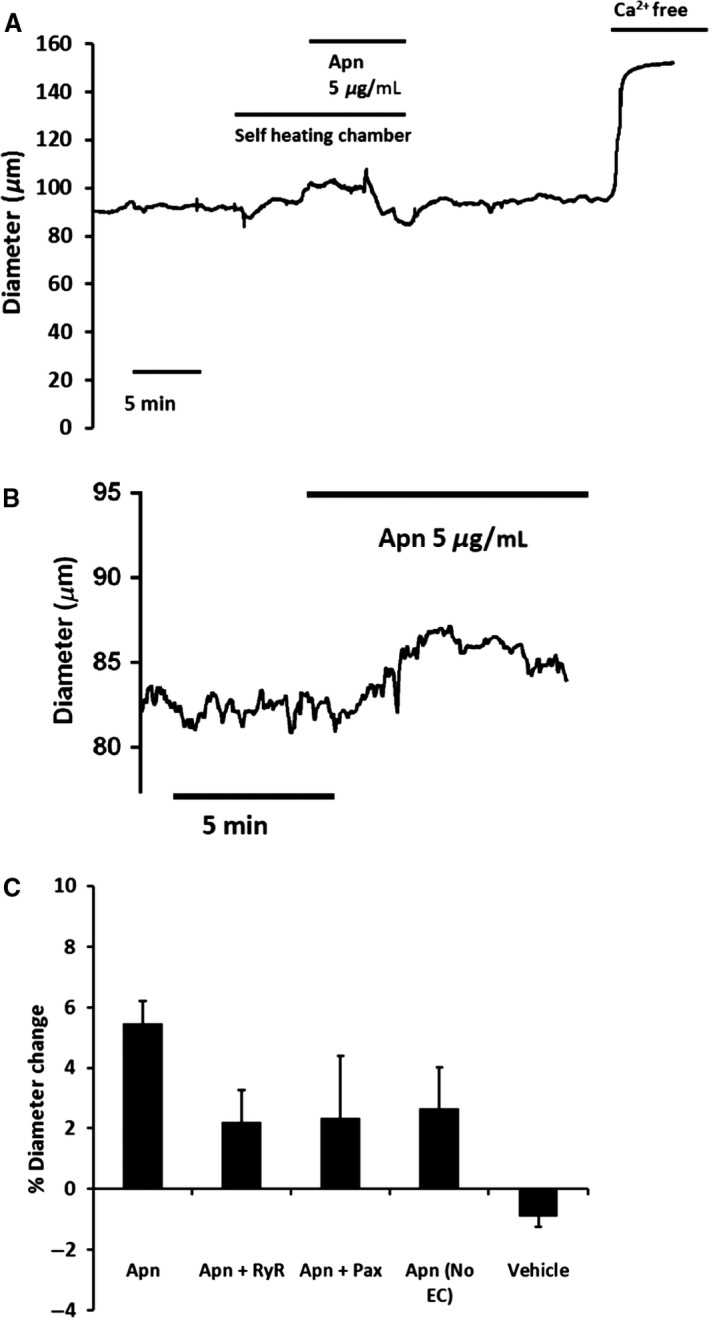
Vasodilation to adiponectin (Apn) was significantly reduced by preincubation with ryanodine (RyR) (20 *μ*mol/L) (Adiponectin 5 *μ*g/mL: 5.5% ± 0.7%, *n* = 6 vs. Adiponectin 5 *μ*g/mL and 20 mmol/L ryanodine: 2.2% ± 1.1%, *n* = 4, *P* = 0.03). The observed changes to the adiponectin vasodilation following incubation with paxilline (Pax) (Adiponectin 5 *μ*g/mL: 5.5% ± 0.7% vs. Adiponectin 5 *μ*g/mL and 1 *μ*mol/L paxilline: 2.4% ± 2%, *P* = 0.11, *n* = 3) or removal of the endothelium (No EC) (Adiponectin 5 *μ*g/mL: 5.5% ± 0.7% vs. Adiponectin 5 *μ*g/mL no endothelium: 2.6% ± 1.4%, *P* = 0.08, *n* = 3) were not significant. (A) Representative trace of adiponectin administered in self‐heating, self‐gassing chamber. (B) Adiponectin administered in self heating chamber onto artery devoid of endothelium. (C) Summary data of vasodilations to adiponectin.

Third order mesenteric arteries constricted with pressure‐induced myogenic tone did vasodilate slightly to adiponectin but the vasodilation was only transient. The maximum point of the vasodilatory change in tone, expressed as a percentage of the overall diameter in Ca‐free PSS (i.e., passive diameter) was similar to that described in wire mounted preconstricted mice mesenteric arteries by Lynch et al. (~6% vasodilation (Lynch et al. 2013)) but less than Fesus et al. who observed a 67% vasodilation of wire mounted arteries. As discussed, previous studies have suggested VSMC BK channels (Lynch et al. 2013) or Kv channels (Fesus et al. 2007) as facilitating mechanisms by which adiponectin manifests vasodilation and also implicated a role for the endothelium (Lynch et al. 2013). Thus, we attempted to examine mechanisms underlying the vasodilation of the pressure constricted arteries to adiponectin by interrupting the Ca^2+^ spark/BK channel pathway and also denuding the endothelium. Any accurate or confident interpretation of the results was limited by the small size of the vasodilation of the pressure constricted arteries, which decreased further following pharmacological intervention. Notably, however, we did not see the increase in adiponectin vasodilation which has been described previously after endothelial removal (Lynch et al. 2013). Nevertheless, the initial vasodilation to adiponectin was reduced by preincubation with ryanodine (20 *μ*mol/L) (Adiponectin 5 *μ*g/mL: 5.5% ± 0.7%, *n* = 6 vs. Adiponectin 5 *μ*g/mL and 20 mmol/L ryanodine: 2.2% ± 1.1%, *n* = 4, *P* = 0.03, unpaired *t*‐test). There were no statistically significant changes to the adiponectin vasodilation following incubation with paxilline or removal of the endothelium. Figure 1A shows administration of adiponectin following the switch from a circulating bath to a self‐heating/air and CO_2_ perfused chamber. Figure 1B is a representative trace of the self‐limiting nature of the vasodilation to adiponectin (in a self‐heating bath).

### Intact PVAT and adiponectin have no effect on Ca^2+^ sparks in VSMCs of mesenteric arteries

**Figure 2:**
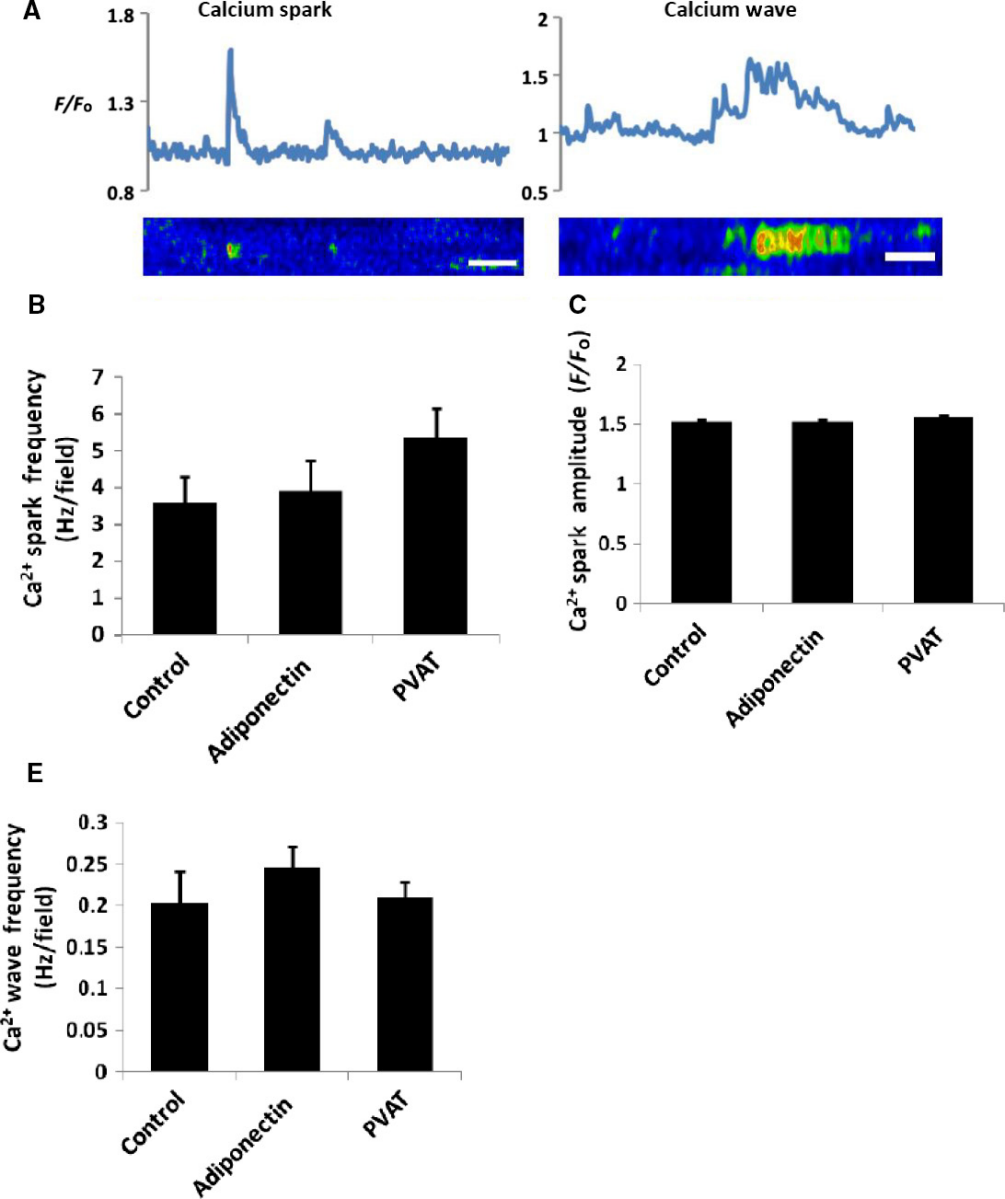
Effect of adiponectin on Ca^2+^ sparks and Ca^2+^ waves. (A) Representative line‐scan traces of changes in fractional fluorescence (F/F_o_) characteristic of Ca^2+^ sparks and Ca^2+^ waves. Neither intact PVAT nor Adiponectin has an effect on Ca^2+^ spark frequency (B), Ca^2+^ spark amplitude (C) or Ca^2+^ wave frequency (D). Line bar represents 0.5s

The regulation of Ca^2+^ sparks and waves by both intact PVAT and adiponectin (5 *μ*g/mL) was studied in 3rd‐order mesenteric arteries. Incubation with adiponectin (5 *μ*g/mL) had no significant effect on spark frequency (3.6 ± 0.7 vs. 3.9 ± 0.8 sparks/field/s before and after adiponectin, respectively; *P* = 0.63, *n* = 10), spark amplitude (1.50 ± 0.01 vs. 1.52 ± 0.01 *F*/*F*_o_ before and after adiponectin; *P* = 0.8, *n* = 10), or wave frequency (0.21 ± 0.04 vs. 0.24 ± 0.03 waves/field/s before and after adiponectin; *P* = 0.16, *n* = 10). Similarly, the presence of intact PVAT had no effect on the small artery Ca^2+^ spark frequency (without PVAT: 3.3 ± 0.7 Sparks/field/second vs. with PVAT: 5.3 ± 0.8, *P* = ns, *n* = 10). The presence of intact PVAT had no effects on Ca^2+^ spark amplitude (without PVAT: 1.52 ± 0.01 *F*/*F*o vs. with PVAT: 1.55 ± 0.02, *P* = 0.357) or Ca^2+^ wave frequency (without PVAT: 0.21 ± 0.04 Hz vs. 0.21 ± 0.02, *P* = 0.91, *n* = 10 Fig. 2).

### Adiponectin increases BK currents in isolated mesenteric artery smooth muscle cells in the absence of Ca^2+^ sparks

**Figure 3:**
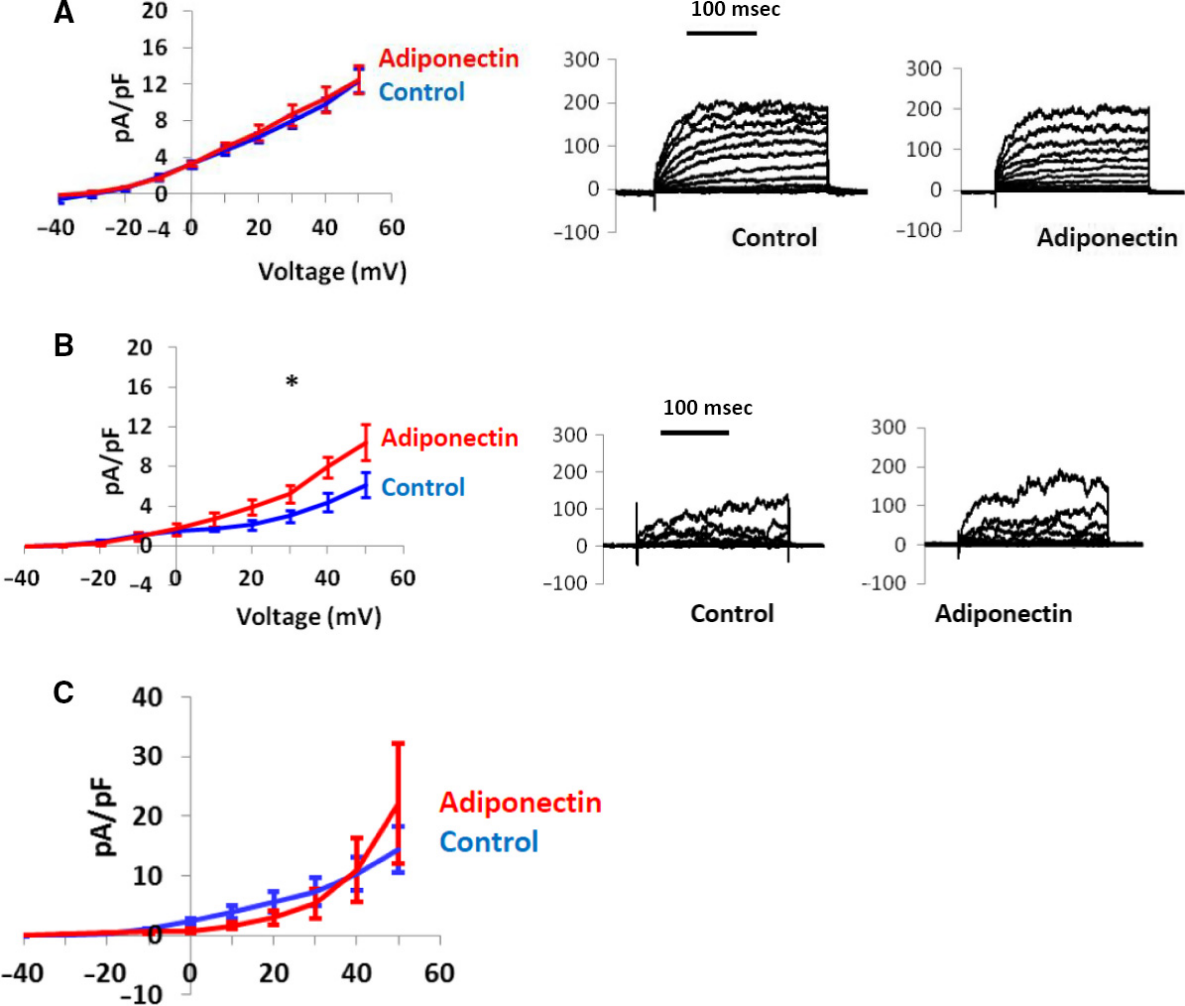
Effect of adiponectin on BK current. Isolated VSMCs were incubated with ryanodine to inhibit STOCs, and the effects of adiponectin on the Kv and BK components of the whole cell outward current were studied using the perforated patch configuration of the whole cell patch clamp technique. (A–B) *Left:* Adiponectin has no effect on the Kv component of the whole cell current (A), but significantly increases BK current (B).(Multiple points ANOVA,* P* = 0.046 for the six data points from 0 mV to 50 mV). *Right:* Corresponding representative traces (C) Isolated VSMCs were isolated and studied as above, but with the traditional whole cell configuration of the patch clamp technique. Using this approach, there was no increase in BK current observed.

To further investigate interactions between adiponectin and VSMC K^+^ channels, we performed patch clamp studies. We first assessed the effect of adiponectin on the K_v_ component of the whole cell K^+^ current, as this had been suggested previously as a potential vasodilating mechanism for adiponectin (Fesus et al. 2007). VSMCs were incubated with both ryanodine (20 *μ*mol/L) to inhibit Ca^2+^ sparks and paxilline (1 *μ*mol/L), a selective BK channel inhibitor. Under these conditions, adiponectin had no discernible effect on the outward current induced by a voltage‐step protocol (−50 mV to +60 mV with incremental voltage steps of 10 mV over 250 msec) (Fig. 3A). To examine the effect of adiponectin on the BK channel current, we incubated cells with ryanodine (20 *μ*mol/L) only and performed a voltage‐step protocol (voltage steps as before) before and after adiponectin. The BK current was then quantified by incubation with paxilline (20 *μ*mol/L) and subsequent offline digital subtraction of the remaining *K*_v_ component. Under these conditions there was a small but significant increase in the BK current after incubation with 5 *μ*g/mL adiponectin (Fig. 3B, multiple points ANOVA, *P* = 0.046 for steps from 0 mV to 50 mV). As outlined earlier, it is thought that in vascular endothelium (Ouchi et al. 2004), and also possibly vascular smooth muscle (Weston et al. 2013), adiponectin mediates effects through activation of intracellular kinases. To investigate whether the observed increase in BK channel current was due to a direct or indirect effect on the channel, we performed experimental protocols using the conventional whole cell configuration of the patch clamp technique rather than the perforated patch configuration, thus dialyzing the cytoplasm of the cell. Using this approach, there was no effect on BK channel current amplitude (Fig. 3C).

**Figure 4:**
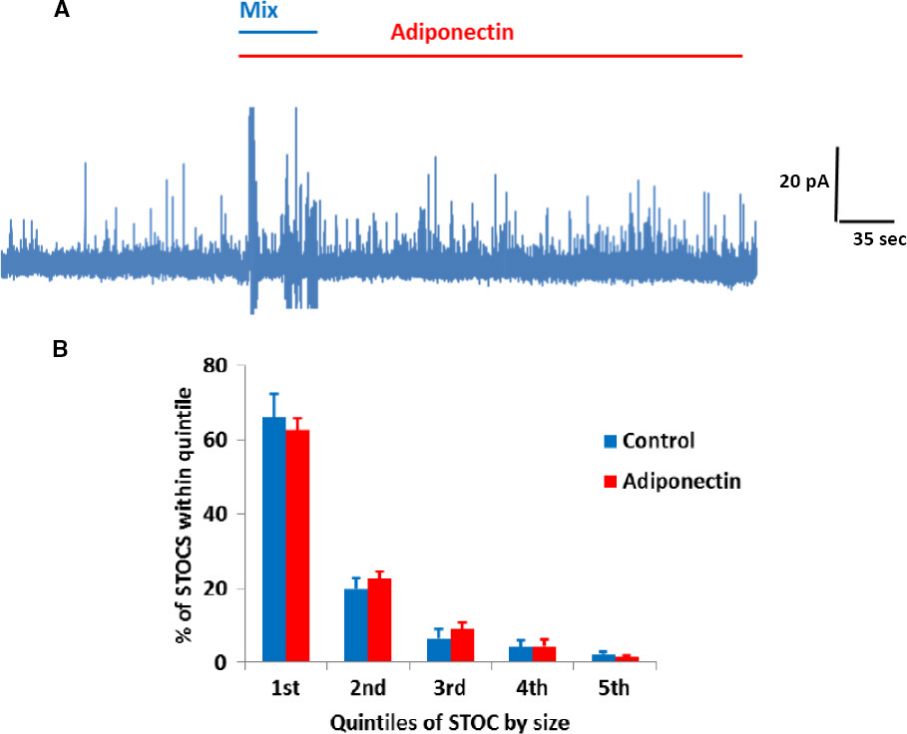
Effect of adiponectin on STOCs. A Representative traces showing STOCs measured during application of adiponectin upon an isolated VSMC. (A) Quantification of adiponectin effect on STOCs in VSMCs (*n* = 5). (B) STOCSs divided into quintiles based on size showing no change in quintile distribution following application of adiponectin.

### Adiponectin has no effect on STOCs

The observed increase in BK channel activity to adiponectin seen using the perforated patch approach and the lack of its effect on calcium sparks suggested that BK‐mediated STOC amplitudes, but not frequency, should increase. However, there was no difference in overall STOC amplitude (15.26 ± 3.3 vs. 18.2 ± 4.1 pA for baseline and adiponectin, respectively; *P* = 0.588, *n* = 5). STOC amplitude does not follow a normal distribution, so we further analyzed the STOC events by dividing STOCs into quintiles of size; an approach similar to that used previously by other studies into the effects on STOC amplitudes of Hydrogen Sulfide (Liang et al. 2012) or Glutamate (Li et al. 2008). However, even adopting this approach, there was no evidence for an effect by Adiponectin on STOC amplitude. Consistent with our observation that adiponectin had no effect on Ca^2+^ sparks, STOC frequency in the presence of adiponectin (82.5 ± 22.3/min) was unchanged compared with baseline STOC frequency (96.6 ± 33.3/min; *P* = 0.734, *n* = 5).

## Discussion

The principal finding from this study was that while adiponectin does have a vasodilatory effect on isolated pressurized arteries, this may not be principally due to a direct interaction between adiponectin and VSMC potassium (K^+^) channels, as previously suspected (Fesus et al. 2007; Lynch et al. 2013). Herein, we present our data to support this position, but also indicate how our findings complement the growing body of work around the vascular effects of this important adipose‐derived cytokine. The actions of adiponectin were studied on small mesenteric resistance arteries constricted with spontaneous pressure‐induced myogenic tone. The vasodilation to adiponectin was small (~5%) although this was consistent with one other group's findings (Lynch et al. 2013). However, neither adiponectin nor the presence of intact PVAT influenced the Ca^2+^ signals which control VSMC large conductance Ca^2+^ activated K^+^ channel (BK channel) activity. Single vascular smooth muscle cell patch clamp protocols suggested that adiponectin had a small effect on the voltage dependent activation of BK channels when the cytoplasm of the cell was intact. However, the increase in current observed through the BK channel following administration of adiponectin was seen only at positive, nonphysiological, membrane potentials. Consistent with this, adiponectin had no effect on the activation of BK channels by Ca^2+^ sparks, manifest as spontaneous transient outward currents (STOCs). Furthermore, adiponectin had no effect on the VSMC K_v_ current.

Adipose‐vascular coupling is the process by which the fat surrounding small arteries (“perivascular adipose tissue” or PVAT) releases vasodilators and thus modulates adjacent small artery contractile tone. In this regard, three main PVAT‐derived vasodilators have been identified: adiponectin (Greenstein et al. 2009; Meijer et al. 2013), hydrogen sulfide (Fang et al. 2009), and nitric oxide (Gao 2007). Our previous work suggests that in human subcutaneous arteries, the majority of the anticontractile effect is mediated by adiponectin (Greenstein et al. 2009; Aghamohammadzadeh et al. 2013). However, in obese patients the ability of PVAT to release adiponectin and thus ameliorate constriction is lost; and this has been proposed as a potential mechanism contributing to the development of obesity related hypertension (Gao 2007). Delineation of the mechanisms by which PVAT and adiponectin modulate contractile tone is therefore important in that it may guide research to identify potential targets for the treatment of obesity‐related hypertension. The rationale for the current study was that previous work suggested that adiponectin exerted its effects on small arteries via VSMC K^+^ channels (Fesus et al. 2007; Lynch et al. 2010; Weston et al. 2013). However, these studies have examined K^+^ channel function in the context of agonist induced constriction of wire mounted arteries and there had been no direct measurement of channel activity using patch clamp or the VSMC Ca^2+^ sparks which modulate their activity under physiological conditions.

Another notable feature of the vascular studies of adiponectin to‐date has been the heterogeneity of the vasodilation to this adipose‐derived hormone. The roughly 5% vasodilation in response to adiponectin of arteries constricted with pressure which we observed was similar to that observed in mesenteric arteries by Lynch et al. (2013) but far less than the 70% vasodilation described by Fesus et al. (2007) in aorta and mesenteric arteries. Furthermore, a recent study has been unable to replicate any vasodilation to exogenous adiponectin of wire mounted rat aorta (even at higher doses) despite using identical protocols (Du et al. 2016) although adiponectin did augment the vasodilation to acetylcholine (Du et al. 2016). An additional consideration is that whilst we saw a small vasodilation to adiponectin, this effect was not sustained. In contrast to Lynch et al., we did not see an augmentation of the vasodilation to adiponectin when the endothelium was removed. We attempted to dissect out mechanisms underlying the vasodilation but any realistic interpretation of our results was limited due to first, the very small and transient nature of the vasodilation which we observed and second the potential of the increase in contractile tone following incubation with ryanodine or paxilline to blunt the subsequent vasodilatory response to adiponectin.

However, a unifying hypothesis to explain the wide heterogeneity of the vasodilatory responses to adiponectin between groups could be that this hormone acts in conjunction with vascular or perivascular structures, particularly perivascular adipose tissue (PVAT) or the perivascular nerves rather than a direct action on the channel. Indeed, a notable finding from Lynch et al. (2013) was that the vasodilation to adiponectin was significantly enhanced when PVAT was left intact. This would be consistent with recent work from Abu Bakar et al. (2017) who showed that electrical field stimulation only dilated mesenteric arteries when PVAT was intact. Furthermore, this study indicated that release of leptin from PVAT secondary to electrical field stimulation contributed to the vasodilation secondary to sensory neurotransmission (Abu Bakar et al. 2017). Also in support of this more integrated PVAT‐nerve‐vessel hypothesis, Weston et al. demonstrated that the *β*3 adrenergic agonist CL316,243 hyperpolarized arteries, but only when PVAT was left attached to the vessel (Weston et al. 2013). Thus, it may be that differences in the preparation of arteries for study are able to account for the heterogeneity of the vasodilation to adiponectin observed between groups. With implications for obesity‐related hypertension, it is pertinent that Abu Bakar et al. (2017) also observed that PVAT release of leptin and adiponectin following electrical field stimulation was attenuated by experimental hypoxia as we have previously seen evidence of hypoxia in functionally damaged PVAT from obese individuals (Greenstein et al. 2009; Aghamohammadzadeh et al. 2013).

The perforated patch clamp protocols used in this study suggested that when the intracellular environment was preserved, incubation of adiponectin increased current through the VSMC BK channel. Conversely, when the cytoplasm of the VSMC was dialyzed using the traditional whole cell configuration of the patch clamp technique, adiponectin had no effect on the BK channel. However, despite the adiponectin‐induced increase in BK channel current seen when cells were studied with a perforated patch‐clamp approach, there was no increase in the frequency or amplitude of the STOCs, suggesting that any effect of adiponectin was not translated into an increase in single cell Ca^2+^ spark induced BK channel activity when the cell was held at a physiological membrane potential. This discrepancy could be accounted for by considering that adiponectin only increased the current through the BK channels of the voltage clamped cells at positive (i.e., nonphysiological) voltages, but it is generally accepted that the rise in Ca^2+^ in the vicinity of a VSMC BK channel secondary to a Ca^2+^ spark maximally activates the BK channel (Perez et al. 2001; Wellman and Nelson 2003). Nevertheless, our diameter and electrophysiological findings do partially concur with those of Weston et al. (2013) who used sharp microelectrode recordings to show that adiponectin hyperpolarizes small mesenteric arteries devoid of endothelium, an effect which was reversed by BK channel blockade. Weston et al. also presented data to suggest that an intermediary activated by adiponectin could be AMP‐activated protein kinase (AMPK), demonstrating that preincubation of the arteries with dorsomorphin (an AMPK inhibitor) partially depolarized the VSMC membrane and also reduced the hyperpolarization to adiponectin. Conversely, an AMPK activator (A769662) caused a hyperpolarization which was reversed by BK blockade (Weston et al. 2013).

AMPK is a ubiquitous kinase which has been traditionally thought to modulate cellular metabolism, although more recently its role has been seen to extend to a broader variety of cellular processes including ion channel function (Bijland et al. 2013; Shirwany and Zou 2010). AMPK is activated by changes to AMP:ATP ratio reflecting metabolic demand, such as hypoxia or ischemia. However, AMPK can also be activated independently of cyclic nucleotides by raising intracellular Ca^2+^, acting via Ca^2+^/calmodulin‐dependent protein kinase and adiponectin has been shown to have this effect in skeletal myocytes (Iwabu et al. 2010). However, the effects of activated AMPK on vascular contractility are not entirely clear, in that different activators of AMPK (AICAR, metformin, phenformin, N_2_‐2DG) show varying effects on diameter. Thus, while AICAR causes a small (~12%) vasodilation, neither metformin nor phenformin have an effect and N_2_‐2DG elicits a vasoconstriction (Rubin et al. 2005). A final consideration is that in our study, neither adiponectin nor the presence of intact PVAT had any effect on calcium signals (Ca^2+^ sparks or waves) in intact pressurized arteries. Nevertheless AMPK is indeed present in VSMC and considering the results from Weston et al. (2013), it is possible that some of the small vasodilation of pressure constricted arteries to adiponectin may be due to AMPK activation independently of Ca^2+^ sparks. However, in regards to arteries constricted with pressure induced constriction, further exploration of this possibility is limited by the small and nonsustained effects of adiponectin.

There are a number of limitations to this study and the interpretations herein. We only used a single concentration of adiponectin (5 *μ*g/mL), but this was equal to or greater than that used in other published studies (Fesus et al. 2007; Lynch et al. 2013; Weston et al. 2013; Du et al. 2016). Although it is difficult to compare results using different experimental approaches on arteries of differing diameters, the vasodilation of pressure constricted arteries to adiponectin was similar to that seen by Lynch et al. (2013) when wire myography of preconstricted arteries was used. We were reluctant to use additional higher concentrations of adiponectin as the 3–5 *μ*g/mL concentration had been shown to be biologically relevant in a number of different functional preparations (wire myography, microelectrode recordings). An exception to these studies has been that by Du et al. when no vasodilation of wire‐mounted preconstricted arteries to adiponectin was seen even at a dose of 15 *μ*g/mL (Du et al. 2016). The experimental approach with a single dose of adiponectin also replicates in vivo conditions. Thus, in healthy human males, adiponectin levels are remarkably stable at around 6‐7 *μ*g/mL, even during fasting (Merl et al. 2005). We acknowledge, however, that serum concentrations of adiponectin in mice are slightly higher (Hashimoto et al. 2013). As discussed earlier, Lynch et al. observed a significant increase in the vasodilatory effect of adiponectin when PVAT was left intact or when the endothelium was removed. Our interventions appeared to have the opposite effect, reducing the vasodilation to adiponectin, even when the endothelium was removed. However, the responses we recorded to adiponectin were both small and transient and thus it was not possible to gain meaningful interpretations from our pressure myography protocols with any realistic degree of confidence. Our single VSMC patch clamp recordings appear to controvert the results of the microelectrode recordings described by Weston et al. (2013) who showed that adiponectin induced a hyperpolarization of intact arteries. However, it is perhaps unrealistic to expect entirely contiguous results when comparing microelectrode recordings from intact arteries against current recordings from voltage‐clamped single cells which also may have been damaged by the digestion process. Nevertheless, interpreting the results from Weston et al. in tandem with the lack of effect of adiponectin that we observed in isolated VSMC may implicate the perivascular nerves in the adiponectin induced hyperpolarization seen in intact arteries.

In summary, however, we have replicated the findings of another group (Lynch et al. 2010) showing that adiponectin at a physiological concentration a mediates a small vasodilation of mesenteric resistance arteries, although this effect was only transient in our preparation. Our results argue against a direct activation of the BK channel by adiponectin, but taken in context with existing work suggests a more nuanced role with involvement of the PVAT or perivascular nerves. An important consideration from our findings is that even though the dilation to adiponectin is small, it would be surprising if a physiological concentration of a hormone were to induce maximal vasodilation of a mesenteric artery constricted with pressure‐induced tone. Furthermore, around 60% to 70% of small arteries (mesenteric, subcutaneous and skeletal) are surrounded by functional PVAT. As such, given that flow through arteries is dependent primarily on diameter, small changes to contractile tone in large numbers of small arteries may have important changes to peripheral resistance and thus blood pressure.

## Conflict of Interest

None declared.
